# Assessment of Small Cellular Particles from Four Different Natural Sources and Liposomes by Interferometric Light Microscopy

**DOI:** 10.3390/ijms232415801

**Published:** 2022-12-13

**Authors:** Anna Romolo, Zala Jan, Apolonija Bedina Zavec, Matic Kisovec, Vesna Arrigler, Vesna Spasovski, Marjetka Podobnik, Aleš Iglič, Gabriella Pocsfalvi, Ksenija Kogej, Veronika Kralj-Iglič

**Affiliations:** 1 University of Ljubljana, Faculty of Health Sciences, Laboratory of Clinical Biophysics, SI-1000 Ljubljana, Slovenia; 2 Department of Molecular Biology and Nanobiotechnology, National Institute of Chemistry, SI-1000 Ljubjana, Slovenia; 3 University of Ljubljana, Faculty of Chemistry and Chemical Technology, Chair of Physical Chemistry, SI-1000 Ljubljana, Slovenia; 4 Institute of Molecular Genetics and Genetic Engineering, University of Belgrade, 11000 Belgrade, Serbia; 5 University of Ljubljana, Faculty of Electrical Engineering, Laboratory of Physics, SI-1000 Ljubljana, Slovenia; 6 University of Ljubljana, Faculty of Medicine, Laboratory of Clinical Biophysics, SI-1000 Ljubljana, Slovenia; 7 Extracellular Vesicles and Mass Spectrometry Group, Institute of Biosciences and Bioresources, National Research Council of Italy, 80131 Naples, Italy

**Keywords:** interferometric light microscopy, interferometric nanotracking analysis, nanoparticle tracking analysis, concentration of extracellular particles, size of extracellular particles, dynamic light scattering, hydrodynamic radius of extracellular particles, extracellular vesicles, liposomes, exosomes

## Abstract

Small particles in natural sources are a subject of interest for their potential role in intercellular, inter-organism, and inter-species interactions, but their harvesting and assessment present a challenge due to their small size and transient identity. We applied a recently developed interferometric light microscopy (ILM) to assess the number density and hydrodynamic radius (R_h_) of isolated small cellular particles (SCPs) from blood preparations (plasma and washed erythrocytes) (B), spruce needle homogenate (S), suspension of flagellae of microalgae *Tetraselmis chuii* (T), conditioned culture media of microalgae *Phaeodactylum tricornutum* (P), and liposomes (L). The aliquots were also assessed by flow cytometry (FCM), dynamic light scattering (DLS), ultraviolet-visible spectrometry (UV-vis), and imaging by cryogenic transmission electron microscopy (cryo-TEM). In R_h_, ILM showed agreement with DLS within the measurement error in 10 out of 13 samples and was the only method used here that yielded particle density. Cryo-TEM revealed that representative SCPs from *Tetraselmis chuii* flagella (T) did not have a globular shape, so the interpretation by R_h_ of the batch methods was biased. Cryo-TEM showed the presence of thin filaments in isolates from *Phaeodactylum tricornutum* conditioned culture media (P), which provides an explanation for the considerably larger R_h_ obtained by batch methods than the sizes of particles observed by cryo-TEM images. ILM proved convenient for assessment of number density and R_h_ of SCPs in blood preparations (e.g., plasma); therefore, its use in population and clinical studies is indicated.

## 1. Introduction

Efforts have been invested to understand the maintenance of cellular homeostasis at the molecular level. However, it has now become clear that the gap between the cellular and molecular levels is too large to be overcome in a single step. It appears that cells communicate in significant ways, including through nanoscaled bits of matter. By virtue of their cell membrane properties, the cells are able to release nanoparticles into the environment, recognize and uptake nanoparticles from the environment, and integrate the material into their substance [1]. Nanoparticles such as extracellular vesicles, protein aggregates, lipid droplets, and viruses can be present in the environment [2] and are suggested to be universal mediators of interaction between life domains [3,4]. To better understand the functioning of cells and organisms, SCPs have been a subject of increasing interest in the last 30 years [5].

By SCPs, we mean sub-micron particles harvested from different samples that contain cells (e.g., conditioned media [6,7,8,9], body fluids [10,11,12,13,14,15,16,17,18,19,20,21], and plant tissues and juices [22,23,24,25]). Acknowledged methods for SCP characterization [26,27] are flow cytometry (FCM) [28,29,30], Dynamic Light Scattering (DLS) [31,32], Nanoparticle Tracking Analysis (NTA) [33,34] and some others, e.g., omics, nucleic acid content [35,36,37,38]. We refer to the methods that take into account a large number of particles as “batch methods”. Of the parameters that can be determined by the batch methods, the most fundamental ones are the number density and the size of the particles in the samples. Different microscopic techniques are being used to determine the morphology of the individual particles in the samples [10,39,40,41]. A golden standard method for characterization of SCPs has not yet been established and complementing different methods is presently the optimal suggested approach.

SCPs prove to be particles with a transient identity that depends on many parameters. The mechanisms of SCPs’ formation remain largely obscure, but it is now generally accepted that the methods used in harvesting and assessing samples may cause profound changes to the particles in them [14,42], in particular those causing mechanical, thermal, or radiation stress on samples [43,44]. Additionally, to validate the methods developed, studies considering a certain number and diversity of samples should be performed, which imposes constraints on the time and cost of the analyses. Minimal invasiveness, cost-and-time effectiveness, and method simplicity are thus issues to consider when designing SCP research. Therefore, efforts are being made to improve the existing methods and develop new ones. 

In liquids, thermal energy causes the random motion of small particles (Brownian motion), and the space within the motion boundaries is inversely proportional to the size of the particle [33,45]. Nanoparticle Tracking Analysis (NTA) employs the Brownian motion of an individual particle by tracking its path with the microscope and analyzing it. However, the resolution of the light microscope is insufficient to directly track SCPs, so light interference was used to improve it [46]. In the recently developed method that is referred to as the Interference Light Microscopy (ILM), the sample is illuminated by a LED light [46]. The focal plane of the objective is imaged on the complementary metal-oxide-semiconductor chip where the source light interferes with the light scattered by a particle located at the objective focal plane [46]. The interference pattern is processed to locate the particle, and a video is recorded to track its movement, from which its diffusion coefficient D is determined. By assuming that the particle is spherical, its hydrodynamic radius R_h_ is estimated by using the Stokes–Einstein relation (see Materials and Methods, Section 4.15). The number density is assessed by counting particles within the volume considered. ILM has recently been introduced into a commercially available instrument, Videodrop (Myriade, Paris, France), and has been used to analyze microorganisms in marine water [47], viruses [48], and extracellular vesicles [49]. 

In this work, we report on the assessment of SCPs from four different natural sources: blood (B), spruce needle homogenate (S), suspension of flagellae of microalgae *Tetraselmis chuii* (T), and conditioned media of microalgae *Phaeodactylum tricornutum* (P), as well as three types of liposomes (L) (made from soya lecithin and water/supernatant of spruce needle homogenate). We isolated SCPs by differential ultracentrifugation from the above natural sources, and we formed liposomes by simple mixing of appropriate quantities of lecithin, glycerol, and water/supernatant of spruce needle homogenate, as described in the Materials and Methods Section. For comparison, the obtained samples were aliquoted and assayed by FCM, DLS, and UV-Vis. We visualized the samples by cryogenic transmission electron microscopy (cryo-TEM). 

## 2. Results

**Figure 1:**
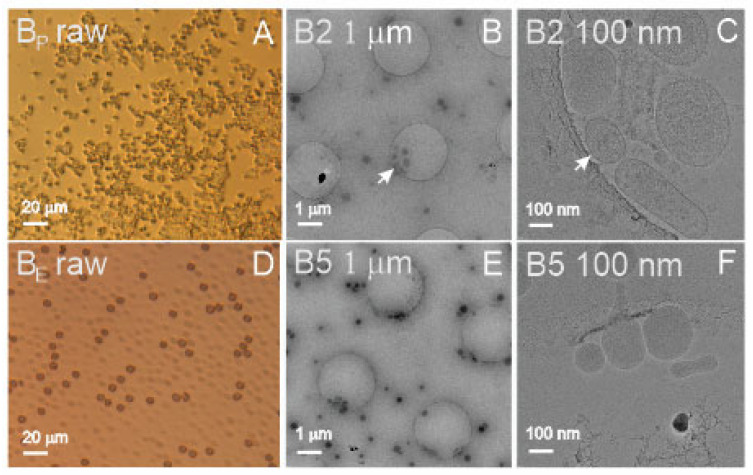
Visualization of blood-derived samples (**A**): blood plasma, (**B**,**C**): isolate from blood plasma, (**D**): suspension of washed, aged erythrocytes, (**E**,**F**): isolate from washed, aged erythrocytes. (**A**,**D**): light microscopy; (**B**,**C**,**E**,**F**): cryo-TEM microscopy. White arrows in panels (**B**,**C**) point to the same spot. Labels in the upper left corner correspond to labels in the raw data (DOI: 10.5281/zenodo.7178202).

Figure 1A shows a light microscope image of blood plasma (B_P_ raw), and Figure 1D shows the suspension of aged, washed erythrocytes (B_E_ raw). It can be seen that the source samples were rich with material (Figure 1, raw). Panel A shows numerous micrometer-sized particles, and Panel D shows numerous erythrocytes (dark particles) and erythrocyte ghosts (transparent particles). In these samples, the SCPs could not be observed by light microscopy as they were too small. Furthermore, the isolates shown in Figure 1 (Panels B,C,E,F) underwent the isolation procedure by ultracentrifugation at high centripetal acceleration of the centrifuge rotor. Figure 1B (B2 1 μm) shows numerous sub-micrometer-sized particles, whereas Figure 1C (B2 100 nm) reveals the presence of different particles, some of them being bilayer-enclosed vesicles with coats of different extensions. Figure 1F (B5 100 nm) shows vesicles isolated from suspension of washed erythrocytes enclosed by a smooth bilayer membrane and an electron-dense interior.

**Figure 2:**
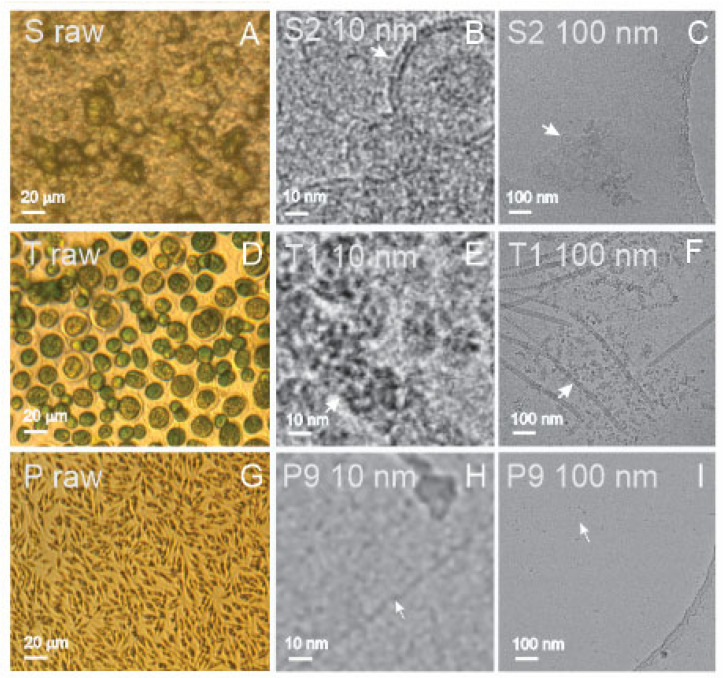
Visualization of samples derived from spruce needle homogenate and microalgae-conditioned media. (**A**): spruce needle homogenate, (**B**,**C**): isolate from spruce needle homogenate, (**D**): culture of *Tetraselmis chuii* microalgae, (**E**,**F**): isolate from *Tetraselmis chuii* conditioned media, (**G**) microalgae *Phaeodactylum tricornutum* culture:, (**H**,**I**): *Phaeodactylum tricornutum* isolate from conditioned media. (**A**,**D**,**G**): light microscopy; (**B**,**C**,**E**,**F**,**H**,**I**): cryo-TEM microscopy. White arrows in panels (**B**,**C**), (**E**,**F**), and (**H**,**I**) point to the same spots, respectively. Labels in the upper left corner correspond to labels in the raw data (DOI: 10.5281/zenodo.7178202).

**Figure 3:**
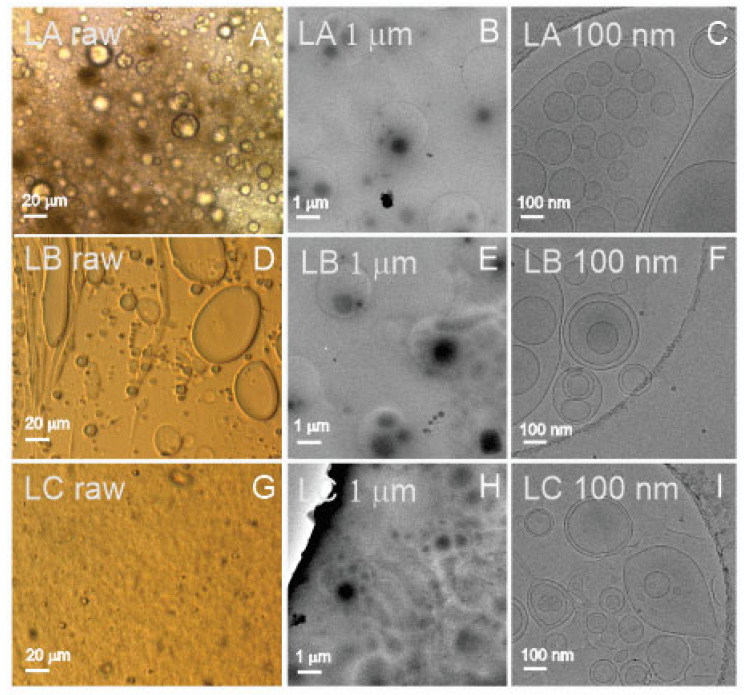
Visualization of liposome samples. (**A**–**C**): 25 weight% of lecithin, 50% of water, and 25% of glycerol, (**D**–**F**): 25 weight% of lecithin, 50% of the supernatant from spruce needle homogenate, and 25% of glycerol, (**G**–**I**): equal weight% parts of lecithin, supernatant from spruce needle homogenate, and glycerol, aged 6 months at room temperature. (**A**,**D**,**G**): light microscopy; (**B**,**C**,**E**,**F**,**H**,**I**): cryo-TEM microscopy. For light microscopy, the samples were undiluted, while for cryo-TEM, the samples were diluted 500×. Labels in the upper left corner correspond to labels in the raw data (DOI: 10.5281/zenodo.7178202).

Spruce homogenate contained remnants of the tissue and residual cells (Figure 2A (S raw)), while the supernatant of the ultracentrifugation contained clusters of small, bilayer membrane-enclosed vesicles with radii below 50 nm (Figure 2C (S2 100 nm) and (Figure 2B (S2 10 nm)). SCPs were also present in the isolate (not shown), but the supernatant after ultracentrifugation presented more vesicles than the isolate. Figure 3D (T raw) depicts a large number of *Tetraselmis chuii* microalgae. Before isolation, the flagellae of the microalgae were removed by a pH shock. The isolate from the flagellae suspension was rich with particles of generally two types of shapes: rods and rosettes (Figure 2F (T1 100 nm)); composed of smaller elements sized about 10–20 nm (Figure 2E (T1 10 nm)); and the rods were of different lengths up to several hundred nanometers (Figure 2F (T1 100 nm)). The source sample of cultured media of *Phaeodactylum tricornutum* was rich with microalgae (Figure 2G (P raw)). In the isolate, we observed small (around 10 nm in size) electron-dense particles of irregular shapes (Figure 2I (P9 100 nm)) that were in some places connected into clusters (not shown). Additionally, we could recognize numerous long, very thin filaments (white arrows) that are poorly visible in Figure 2I (P9 100 nm) but recognizable at higher magnification (Figure 2H (P9 10 nm)). 

Figure 3 shows images of three types of liposomes: LA (25 weight% of lecithin, 50% of water, and 25% of glycerol), LB (25% of lecithin, 50% of supernatant from spruce needle homogenate, and 25% of glycerol), and LC (equal weight% parts of lecithin, supernatant from spruce needle homogenate, and glycerol; liposomes aged 6 months at room temperature). The samples, which present as a viscous and very dense brownish paste, were smeared on cover glass to form a thin layer. These samples could be imaged directly by light microscopy (assigned “raw”, Panels A,D,G in Figure 3). All three samples (LA raw, LB raw, and LC raw) contained particles of different sizes, from several micrometers down to the resolution threshold of light microscopy (Figure 3 raw). It can be seen that the samples were very rich in particles. To visualize the samples by cryo-TEM and measure them by other methods, they were diluted by ultraclean water as indicated in Table 1. Cryo-TEM images (Figure 3C,F,I (LA 100 nm, LB 100 nm, and LC 100 nm)) show the presence of vesicles enclosed by a bilayer membrane with effective radii from hundreds of nanometers down to 50 nanometers. The micrometer-scale images (Figure 3B,E,H (LA 1 μm, LB 1 μm, and LC 1 μm)) show an abundance of liposomes in the samples. All three samples (LA, LB, and LC) appeared similar: rich with vesicles of different sizes, the small ones being the most abundant ones. We observed no differences between the sample LC, which was aged for 6 months at room temperature, and the fresh samples LA and LB. 

Table 1 shows the results of measurements on the samples from four natural sources: blood (B), spruce homogenate (S), flagellae of *Tetraselmis chuii* (T), conditioned culture media of *Phaeodactylum tricornutum* (P), and liposomes (L), by four methods (FCM, ILM, DLS, and UV-vis). In FCM, we assessed the number of events in the scatter diagram. In ILM, we assessed the number of particles (N) per mL. In DLS, we assessed the intensity of scattered light I for the peaks in the I(R_h_) distribution, where R_h_ is the hydrodynamic radius of the particles. We detected two–four peaks in the I(R_h_) distribution. In UV spectroscopy, we detected the absorption of light at wavelength 280 nm likely due to proteins, while in samples of blood, we also detected the absorption of light at a wavelength of 420 nm which was assigned particularly to the presence of hemoglobin. In some cases, it was not possible to apply all the methods to all samples, either because there was not enough material available or because there were technical difficulties with the measurement. Empty spaces in Table 1 mean that the sample was not analyzed by the respective method. 

In blood-derived samples, we did not perform FCM measurements of the isolate from plasma due to the small amount of the sample. There were no difficulties in the assessment of the blood-derived samples by either of the methods. The particle number density was about four orders of magnitude higher in the ILM measurement than in the FCM measurement. The difference was the largest in the isolate from washed erythrocytes (B5). The isolate was found to be rich with small vesicles (Figure 1E, B5 1 μm) which are not likely to be detected by FCM. In DLS measurements, the scattering of light could be clearly detected. In addition to the 280 nm wavelength, UV-vis absorption was examined at 420 nm, where the signal was expected to reflect the presence of hemoglobin. The highest absorption at 420 nm (13.8 mg/mL) was confirmed in sample B1 (washed erythrocytes). 

Isolated SCPs from spruce homogenate could not be analyzed by DLS (Table 1). However, particles were detected by FCM and ILM in some samples. Moreover, the absorption by UV-vis in all samples from spruce homogenate was high (Table 1), indicating the presence of proteins in the samples. Small colloidal vesicles adhering to each other in the form of clusters were observed in the supernatant after ultracentrifugation by cryo-TEM (Figure 2B, S2 10 nm, and Figure 2C, S2 100 nm).

Flagellosomes were detected in the isolate from a sample of harvested flagellae derived from conditioned culture media of the microalgae *Tetraselmis chuii* by FCM and ILM (Table 1, sample T1). However, the absorbance at 280 nm measured by UV-vis in this sample was very low (Table 1). Cryo-TEM images revealed the structure of the particles: nano-sized rods and rosettes composed of even smaller units. These structures were previously identified as scales and hairs of *Tetraselmis chuii* flagellae [50]. The presence of particles was detected and analyzed by ILM and DLS by applying the Stokes–Einstein relationship, based on the assumption that the particles in the samples are spherical (Table 1). 

The isolate (a pellet of NPs obtained after differential ultracentrifugation) obtained from the conditioned media of the microalgae *Phaeodactylum tricornutum* was not visible to the naked eye. The isolated SCPs could not be analyzed by DLS (Table 1). The protein content as measured by UV-vis spectroscopy was low in all samples (Table 1). However, both FCM and ILM detected the presence of particles in all samples that were analyzed (Table 1). By cryo-TEM, about 10 nm-sized electron-dense particles of irregular shapes were observed (Figure 2I, P9 100 nm), occasionally connected into net-like clusters (not shown), and many long, very thin filaments (Figure 2H, P9 10 nm). In some samples from *Phaeodactylum tricornutum*, particles larger than 1 μm were observed by ILM; moreover, bacteria were detected in some samples, (Table 1). 

Liposome samples were prepared in two dilutions (100× and 1000×; c.f. the dilution factor in column 2 in Table 1) to suit measurements with DLS. All samples were filtered before aliquoting through a 0.4 μm filter. The measurement of liposomes presented no difficulties in either of the methods. In DLS, all samples were found to be homogeneous. In FCM, only 1000× diluted samples were measured, as the 100× diluted samples were lost in the process. In ILM, the samples were additionally diluted to suit the method as given in Table 1. In cryo-TEM, the samples were additionally diluted 5×.

In liposomes, the number of events detected by FCM was about five orders of magnitude smaller than the number of particles detected by ILM. As the limit of detection of a flow cytometer is about 400 nm and the majority of particles are expected to be smaller than this limit, which in turn can be exceeded by ILM, the difference in the detected number of particles by FCM and ILM seems reasonable. 

While FCM and ILM measure the number of particles, DLS and UV-vis detect quantities that are proportional to the number of particles but involve other parameters. The order LC > LA > LB was obtained in 1000× diluted liposome samples by FCM, UV-vis at 280 nm, and in 100× diluted samples at 280 nm (Table 1). 

Proportions of amounts detected in liposome samples diluted 1000× and 100× were assessed by ILM, DLS, and UV-vis and were within the expected errors of the methods (Table 2). DLS and UV-vis were subjected to smaller biases than ILM, as for ILM the samples had to be additionally diluted, causing changes in the self-assembly of liposomes. The results of DLS and UV-vis are given in proportion to the number of particles. 

The quantity that reflects the size—the hydrodynamic radius R_h_—was determined by ILM and DLS. The correlation function was used to fit the DLS data, which resulted in I(R_h_) distributions with up to four peaks and four corresponding mean R_h_ values of the populations (Table 3: the R_h_ value of the largest population exceeded 10 µm and is not reported in Table 3). Particle sizes in most samples were between 100 and 200 nm, according to ILM (Table 3). This agreed well with the DLS results (compare with the R_h_ value in Table 3 that corresponds to the main peak in the I(R_h_) distribution, i.e., to the peak with the highest contribution to I). In the isolate from plasma (B3), smaller R_h_ were assessed by both ILM and DLS (68 and 39 nm, respectively), indicating the presence of a large proportion of lipoproteins and protein complexes. As DLS measurements of liposomes and blood-derived vesicles indicated more than one peak, a wide distribution of particles with respect to size was indicated; however, the small intensity of the peaks at larger R_h_ suggested that the majority of the particles were small. The presence of particles with R_h_ between 100 and 200 nm was recorded in isolates from flagellae of the microalgae *Tetraselmis chuii* by ILM; however, globular particles of that size were not predominant in the cryo-TEM images (Figure 2F, T1 100 nm). Instead, cryo-TEM revealed many non-spherical particles in the isolate (Figure 2F, T1 100 nm, and Figure 2E, T1 10 nm). The presence of particles with effective radii between 100 and 200 nm was also recorded in isolates from conditioned media of *Phaeodactylum tricornutum*, while the cryo-TEM showed mostly 10 nm electron-dense particles (Figure 2I, P9 100 nm) and thin filaments (Figure 2H, P9 10 nm). 

## 3. Discussion

We have assessed 30 samples by three acknowledged batch methods (FCM, DLS, and UV-vis) and a recently developed method (ILM) and visualized them by cryo-TEM. The batch methods provide information on a sample containing a large number of particles. We determined the amount of SCPs by the number of events recorded by FCM in the FSC/SSC scatter diagram, counting individual particles in a volume of suspension by ILM, by measuring the intensity and fluctuations of scattered light by DLS, and measuring the absorbance of light by UV-vis spectroscopy. We estimated the size of SCPs by analyzing the correlation function with DLS and by estimating the region of the Brownian motion with ILM. We could visualize the size of some SCPs in cryo-TEM images. 

Erythrocyte vesicles have been visualized before by SEM, TEM, and cryo-TEM [20,51,52], and the SCPs observed in our samples agree with previous reports. Cryo-TEM images show bilayer membrane-enclosed globular colloidal vesicles with effective radii between 50 and 100 nm containing an electron-dense interior—probably aggregated hemoglobin [53,54]. Light microscopy of sample B1 revealed washed erythrocytes and ghosts averaging about 7 µm in diameter of the circular contour (Figure 1A, BE raw), which were also spotted in ILM (but not included in ILM’s determination of particle number density and size) and assessed by FCM. The sample B1 also contained many SCPs, as measured by ILM (Table 1). Sample B2 contained the isolate from plasma. The quantity of the isolate from a 2.7 mL tube of blood was of the order of 25 μL. Although the isolate was diluted with 80 μL of PBS, the quantity was too small to perform the measurements by all four methods. We decided to omit measurement by FCM, which would require a considerable portion of the sample. Similarly, erythrocyte vesicles in B2 had smooth shape contours corresponding to the minimum of membrane free energy [52], whereas the membrane in some vesicles had a coat that could have been formed by adhesion of molecules from plasma (i.e., the protein corona) (Figure 1C, B2 100 nm). Previous observations of plasma by cryo-TEM indicate that platelets may fragment to release different kinds of SCPs [40,41,55], whereas these SCPs are in the size range of the ILM. 

Samples B3 and B4 were the supernatants of the centrifugation of plasma at 17,570 g, the first one representing platelet-poor plasma and the second one representing PBS, with which the isolate was washed. B3 exhibited the smallest SCPs (68 nm on average as estimated by ILM and 39 nm corresponding to the main peak as estimated by DLS). Plasma contains many different types of SCPs, including lipoproteins sized 5–80 nm [40]. The results of all four methods agree that sample B4 contained the fewest cell-sized particles (FCM), the fewest particles in the size interval 100–1000 nm (ILM), the fewest particles that scatter light (DLS), and the fewest proteins (UV-vis) compared to other blood-derived samples. Samples B5, B6, and B8 were isolated from in vitro-aged, washed erythrocyte suspensions. Sample B5, which was imaged by cryo-TEM (Figure 1B, B5 1 μm, and B5 100 nm), had the largest number of SCPs as determined by FCM and ILM. Samples B6 and B8 were not assessed by DLS due to the small quantity of the samples.

To the best of our knowledge, cryo-TEM was the first to detect colloidal vesicles from spruce needle homogenate (Figure 2B,C, and S2 100 nm). Singular vesicles were also observed in the isolate (not shown). The particles appeared as small bilayer membrane-enclosed vesicles (Figure 2B, S2 10 nm) that adhered to each other to form clusters (Figure 2C, S2 100 nm). In the process of homogenizing spruce needles, we saw the formation of rich green foam, indicating that the samples contained molecules that are prone to form vesicles during the harvesting and assessment processes. The UV-vis absorption at 280 nm (Table 1) indicated that the samples were rich with proteins, which mediate the bridging interaction between the membranes [56]. We were not able to detect the presence of SCPs by DLS since the samples contained larger particles, most probably the remnants of the homogenization, which caused strong scattering of light. The vesicles observed by cryo-TEM were smaller than 100 nm (Figure 2C, S2 100 nm), but formed clusters that are expected to be moving synchronously in Brownian motion and were therefore detected as such by ILM.

Microalgal SCPs present a challenge as the amount of material in the isolate is small. We assumed that the yield of SCPs would be higher from the samples containing harvested flagella of *Tetraselmis chuii*. *Tetraselmis chuii* has four flagella that are released when the microalgae are stressed; however, the microalgae may grow new flagella in a time interval of several hours. We have created a pH shock by adding HCl to the conditioned culture media to deflagellate the microalgae. Flagella were then separated from the cells by centrifugation, and SCPs were isolated from the suspension. Cryo-TEM revealed that the representative SCPs in these isolates were intrinsically different from colloidal vesicles from blood preparations, liposomes, and preparations from spruce needle homogenate. Their shape and structure were not that of a bilayer membrane that encloses a structureless interior; we observed rod-like structures and ordered complexes of about 10 nm elements (Figure 2E, T1 10 nm). The UV-vis absorption at 280 nm was weak, in line with previous results that the flagella scales were mostly composed of carbohydrates [50]. The shape of these flagellosomes was not globular; therefore, they do not comply with the assumptions underlying the analysis of motion and scattering by ILM and DLS, respectively. Although the results of ILM and DLS indicate that particles are present in the samples and give values for their estimated R_h_ (Table 3), it is clear from the cryo-TEM image (Figure 2F, T1 100 nm) that these particles are not bilayer-enclosed vesicles with globular shapes. Without visualization of the samples, considering only the results of the batch methods would lead to misleading conclusions that the sample contained globular particles with an effective radius of ~120 nm (ILM)/53–70 nm (DLS) (Table 3).

To isolate SCPs from *Phaeodactylum tricornutum*, we started with a relatively large volume of the culture and collected the isolates in about 30 P samples. In none of the samples could we observe an isolate by eye. UV-vis results indicated low protein content (Table 1), and DLS measurements were encountered with difficulties: weak scattering on small particles and the presence of a few large objects that scattered strongly and thus obstructed the measurement. We imaged one of the isolates (P9) by cryo-TEM, in which we found no membrane-enclosed vesicles or other types of vesicles of several hundred nanometers; however, there were many small (about 10 nm) electron-dense particles occasionally connected into net-like clusters and many long thin filaments (Figure 2I, P9 100 nm, and Figure 2H, P9 10 nm). We could detect particles by FCM and ILM in all samples (average R_h_ over all samples: 175 nm) and by DLS in two P samples (P3 with R_h_ = 170 nm and P4 with R_h_ = 190 nm; Table 3). R_h_ in both ILM and DLS is determined by the Stokes–Einstein equation (see Material and Methods, Section 4.14), and is inversely proportional to the diffusion coefficient D. In turn, D is proportional to the square of the velocity of the particle [46]. As smaller and lighter particles move faster, they have a larger D and therefore a smaller R_h_. In Brownian motion, the particles are constrained only by collisions with their neighbors; however, the presence of thin filaments in P samples may pose additional constraints to the movement of the particles and lower/increase their measured D/R_h_. The presence of thin filaments indicates that the obtained R_h_ was overestimated, because the particles moved more slowly before becoming trapped in the filaments rather than because they were larger.

Cryo-TEM revealed that samples L were rich with liposomes (sample LA contained water while samples LB and LC contained supernatant of isolation from spruce needle homogenate) appearing as spherical or elongated colloidal vesicles in which a bilayer membrane enclosed a fluid interior. The size spanned three orders of magnitude (Figure 1). We found no other type of particle in the imaged samples (Figure 1). As the starting material of the samples was a viscous and very dense brownish fluid, the samples had to be diluted for measurements. We prepared two sets of samples (at 100× and 1000× dilution), which were appropriate for the DLS assessment. All the diluted samples appeared to be transparent, colorless, but slightly turbid solutions. As the particles appeared globular in cryo-TEM, the theoretical background for ILM and DLS was justified. The samples were also measured by UV-vis. Only 1000× diluted samples were measured by FCM, while the required quantity of 100× diluted samples was lost in preparation. The samples were additionally diluted for the measurement with ILM as well as for imaging with cryo-TEM (Table 1). As the proportion of lipid, water, and glycerol is crucial in the determination of the shape and size of liposomes, it is likely that additional dilution has changed the identity of the liposomes and that different methods have actually measured different particles with respect to size. Furthermore, in FCM, the sample is subjected to flow, which may deform the liposomes and cause their fragmentation. The numbers of liposomes per mL measured by FCM and ILM were different. The reason could be that, besides different dilutions of the samples, the instruments detect particles in different size ranges, which are also present at different number densities.

Direct assessment of the size among the methods used is possible only by Cryo-TEM, but it should be considered that vesicles larger than the thickness of the ice are squeezed and that endovesiculation can occur in the process of vitrification [57]. Some evaporation may take place during the preparation of the specimen, and in a thin liquid film, a significant local increase in solute concentration may take place. It was suggested that just before vitrification, the lipid vesicles may adjust by losing water while practically no ions have time to cross the bilayer [57]. The volume of the vesicle decreases while its surface remains constant, and the vesicle may form an invagination that progresses to concentric vesicles.

The R_h_ of liposomes was estimated by ILM and DLS (Table 3); in ILM, the reported sizes were in the range between 137 and 152 nm, while in DLS, the interval of the main peak was between 122 and 235 nm (Table 3). These sizes were within the observations by cryo-TEM (Figure 3). DLS detected peaks at both smaller and larger R_h_ values, indicating that the size distribution of liposomes was broad.

Additionally, other authors presented results of the studies where they used different characterization methods on the same samples. Van der Pol et al. determined the particle size distribution of standard polystyrene beads and standard SCPs from urine by TEM, flow cytometry, NTA, and resistive pulse sampling (RPS) [58]. They claimed a different size distribution between the methods used; however, as for determination of the size, they considered assessment by TEM as a reference. The differences in results obtained by different methods were assigned to differences between the minimum detectable vesicle sizes (NTA: 70–90 nm, RPS: 70–100 nm, specialized FCM: 150–190 nm, FCM: 270–600 nm). Maas et al. compared EP quantification using three different detection techniques: NTA, RPS, and an optically optimized high-resolution FCM [59]. They analyzed cell-derived SCPs and liposomes of known sizes. They found larger differences in quantification for liposomes compared to SCPs using all three techniques. Comparable results regarding the quantification of SCPs were found for RPS and FCM when fluorescence-based triggering was employed.

Assessment of EP isolates is challenged by the small size, fragility, and transient identity of the particles, the variability of their number density and refractive index, and their heterogeneity in composition and morphology. It is generally agreed that consensus and standardization are crucial to being able to compare scientific data and collaborate to achieve a better understanding of the mechanisms of EP formation and biological roles [29,30,60,61]. Presently, a combination of methods is a suggested way to approach these challenges [62]. However, improvements to the existing methods and the development of new ones are also pursued.

In addition, to the recently developed ILM presented here, an attempt has been made to improve NTA resolution by including interference from incident laser light and light scattered by the SCPs. Kaskhanova et al., 2022 [45] reported on the improved resolution with respect to classical NTA by interferometric nanotracking analysis (iNTA) with which they were able to distinguish the polystyrene beads of different sizes and assess samples with heterogeneous nanoparticle composition [45]. Although this technique shares some of the same physical phenomena as ILM (interference between the scattered and the incident light, nanotracking), it differs in illumination (iNTA employs laser light, which has higher energy to reach a lower limit of detection, whereas ILM employs LED light) and optical configuration (iNTA uses refracted light, whereas ILM uses direct light). Furthermore, iNTA was recently presented at the research results level [45], while ILM is already commercially available.

A variety of methods are used to isolate SCPs, each with their advantages and limitations. Ultracentrifugation/gradient ultracentrifugation separates particles by their sedimentation speed subject to centrifugal acceleration; size exclusion chromatography uses columns that separate SCPs larger than some threshold from smaller particles; and SCPs can be sorted by filtration [6,7,11,13]. These methods involve mechanical manipulation of SCPs and, to some extent, change their number, size, shape, and composition. Additionally, characterization methods impact SCPs; they may require dilution or filtering of samples, which can change their number, size, and morphology; FCM and resistive pulse sensing impose shear stress in the flow; methods based on labeling may change the composition and morphology of SCPs, etc. Additionally, SCPs may be lost in the process due to sticking to the material that comes in contact with the samples (pipette tips, tubes, etc.). Different methods need different quantities of the sample. Additionally, there is a difference in time and cost and in the required skills to use the method, which may be key to the study design.

Low invasiveness of the isolation and characterization methods is advantageous. ILM can detect particles in the range of approximately 50–800 nm. The presence of larger particles (cells) does not prevent the measurement, so samples do not need to undergo harsh “purification” and filtration procedures. DLS, nano-FCM, and resistive pulse sensing (RPS) methods can also detect particles smaller than 50 nm. The advantage of ILM with respect to (nano) FCM and RPS is that the sample does not enter the instrument (e.g., tubes), and therefore larger particles cannot clog them. DLS measurements are disturbed by larger particles, and these particles should be removed. This is often done by filtration. However, as SCPs are dynamic systems, they may change considerably due to the mechanical procedures involved in “purification” and filtering.

Both DLS and ILM are optical methods and are minimally invasive to the samples. DLS requires about 100 μL of an appropriately diluted sample, while ILM requires about 7 μL of an appropriately diluted sample. DLS can also detect very small particles down to nanometer sizes, while ILM is limited to about 80 nm, depending on the other properties of the sample. In our samples, ILM yielded an average R_h_ of 69 nm in plasma, which agreed reasonably well with the 39 nm detected by DLS (Table 3). Furthermore, in T1, DLS detected a peak at about 20 nm that could not be detected by ILM (Table 3). Although the determination of R_h_ (by both DLS and ILM) is not a relevant method to assess the non-spherical particles, DLS (but not ILM) indicated the presence of smaller particles in the samples. On the other hand, the presence of particles larger than about 1 μm disturbed the measurements with DLS but not with ILM (see raw data, sample B1). DLS measurement of isolates from *Phaeodactylum tricornutum* was successful in only two samples due to the presence of larger particles in other samples, while ILM gave results for all samples. DLS required on average about half an hour per sample for the measurement and analysis by a highly skilled researcher, while ILM required about 10 min.

Cryo-TEM revealed colloidal vesicles in the isolate from plasma (Figure 1C, B2 100 nm) with a number density of 1.8 × 10^10^/mL (determined by ILM) and R_h_ 115 nm (ILM)/185 nm (DLS). The average effective radius of the vesicles measured from Figure 1B, B2 1 μm (30 vesicles) was 118 ± 49 nm. The isolate from washed erythrocytes contained colloidal vesicles (observed by cryo-TEM) with a number density of 1.8 × 10^11^/mL (ILM) and R_h_ = 106 nm (ILM) or 153 nm (DLS). The effective radius of the vesicles measured from Figure 1E, B5 1 μm (62 vesicles) was 112 ± 31 nm. UV-vis results indicated that the isolate from plasma (B2) contained more proteins than the isolate from washed erythrocytes (B5) (based on measured absorbance at 280 nm), while the isolate from erythrocytes contained hemoglobin (based on measured absorbance at 420 nm). The standard deviation of the vesicles’ sizes measured from cryo-TEM images was larger in the isolate from plasma than in the isolate from erythrocyte suspension (49 vs. 31 nm) (raw data). Additionally, the standard deviation of R_h_ measured by ILM was larger in the B2 sample than in the B5 sample (123 vs. 55 nm) (raw data). This indicates that SCPs isolated from plasma (B2) were more heterogeneous in origin than SCPs isolated from erythrocyte suspension (B5).

Using cryo-TEM, we identified for the first time small colloidal vesicles with radii under 100 nm in the supernatant of spruce homogenate (Figure 2C, S2 100 nm), while the material could not be analyzed by DLS and was below the detection limit of ILM. In the isolate from spruce homogenate (S9), a number density of 2.82 × 10^8^/mL and the average R_h_ (=106 nm) of SCPs could be assessed by ILM (but not DLS) (Table 1 and Table 3, respectively).

Both ILM and DLS detected flagellosomes isolated from suspension of *Tetraselmis chuii* flagellae and their number density was assessed by ILM (9.6 × 10^8^/mL) (Table 1). UV-vis indicated that flagellosomes were poor in protein content. Cryo-TEM showed that the particles were not spherical and therefore did not comply with the Stokes–Einstein equation, which is based on the motion of spherical particles. It would therefore be more appropriate to present the results in terms of the diffusion coefficient that relates to the movement of the particles.

In isolates from conditioned culture media of *Phaeodactylum tricornutum,* the number density (2–6 × 10^8^/mL) and R_h_ (140–200 nm) of SCPs could be assessed by ILM and DLS (180–190 nm) (Table 3). However, we did not observe particles of that size by cryo-TEM (Figure 2I, P9 at 100 nm). The majority of particles were about 10 nm-sized electron-dense particles embedded in a background of thin filaments (Figure 2I, P9 100 nm). A possible explanation of this disagreement between ILM and DLS on one side and cryo-TEM on the other is the restrained motion of particles by numerous thin filaments in the samples (Figure 2H, P9 10 nm), which leads to an overestimation of R_h_.

Sample L consisted primarily of bilayer-enclosed globular liposomes (with no other particles) ranging in size from thirty nm to hundreds of μm. From these, the majority were small (as deduced from FCM, ILM, and DLS). The number density of liposomes was about 1 × 10^14^/mL (average over all ILM results), and their average R_h_ was 160 nm (average over all ILM (150 nm) and DLS (170 nm) results, Table 1). Samples were rich with proteins (Table 1), and there were no apparent differences between liposomes made with spruce supernatant or ultraclean water or between fresh liposomes and six-month-aged liposomes at room temperature (UV-vis).

SCPs would be interpreted as spherical particles of the given R_h_ if only the batch methods ILM and DLS were used. This agrees well with the images of colloidal vesicles observed in B and L samples (Figure 1 and Figure 3). In S samples, cryo-TEM showed that the particles were not colloidal vesicles of the given size but clusters of smaller colloidal vesicles (Figure 2C, S2). In the T sample, cryo-TEM showed that flagellosomes differ considerably in shape from spherical particles (Figure 2F, T1), and in the P sample, it indicated that the properties of the medium in which the particles moved may have caused the apparent overestimation of R_h_. Visualization is thus key to a realistic assessment of the samples.

## 4. Materials and Methods

### 4.1. Chemicals

Edible soya lecithin was from Fiorentini, Torino, Italy.

### 4.2. Blood Sampling

Blood was donated by an author (a female with no record of disease). Collection was established in the morning after fasting for a minimum of 12 h overnight. A G21 needle (Microlance, Becton Dickinson, Franklin Lakes, NJ, USA) and 2.7 mL evacuated tube with trisodium citrate (BD Vacutainers, 367714A, Becton Dickinson, Franklin Lakes, NJ, USA) were used.

### 4.3. Preparation of Washed Erythrocytes and Plasma

To sediment the erythrocytes, blood was centrifuged for 10 min at 300× *g* and 18 °C (centrifuge Centric 400/R, Domel, Železniki, Slovenia). The supernatant from above the buffy coat was collected into Eppendorf tubes to constitute 250 microliter plasma aliquots. The erythrocytes were resuspended in PBS by turning the eprouvette upside-down and centrifuged for 10 min at 300× *g* and 18 °C (centrifuge Centric 400/R, Domel, Železniki, Slovenia). The supernatant was removed and replaced by PBS. Erythrocytes were resuspended by turning the eprouvette upside-down and centrifuged for 30 min at 500× *g* and 18 °C (centrifuge Centric 400/R, Domel, Železniki, Slovenia). The washed erythrocytes were stored in buffer solution at 4 °C. 

### 4.4. Cultivation of the Algae

Cultures of *Tetraselmis chuii* CCAP 66/21b and *Phaeodactylum tricornutum* CCAP 1052/1A from the Culture Collection of Algae and Protozoa (CCAP) of SAMS (Oban, Scotland) were grown in artificial seawater (Reef Crystals, Aquarium Systems, Sarrebourg, France). Twenty-two grams of salt were dissolved in one liter of distilled water, sterile filtered (0.2 micron cellulose filters, ref. 11107-47-CAN, Sartorius Stedim Biotech GmbH, Gottingen, Germany), autoclaved, and supplemented with Guillard’s (F/2) Marine Water Enrichment Solution (ref. G0154, Sigma Aldrich, St. Louis, MO, USA). Cultures were grown in 0.5 L borosilicate bottles in a respirometer (Echo Instruments, Slovenske Konjice, Slovenia) at 20 °C and 20% illumination with a 14 h light/10 h dark cycle and an aeration rate of 0.2 L/min.

### 4.5. Preparation of Spruce Needle Homogenate

Branches were cut from the *Picea abies* tree and used immediately. Branches were immersed in 1.5 L of water at 30 °C with 10 mL of sodium hypochlorite (NaClO, 0.1%) for 1 h. The branches were rinsed with water. The needles were cut off from the branches. 50.0 g of wet needles were immersed in 300 mL of ultraclean water and stirred for 1 min in a KOIOS 850W Smoothie Bullet Blender (KOIOS, Neweg, City of Industry, CA, USA). The homogenate was filtered through 0.5 mm nylon net cloth to remove larger particles.

### 4.6. Isolation of Flagellosomes

*Tetraselmis chuii* microalgae were harvested when a stable logarithmic growth phase was reached (about one month after inoculation). Flagella were removed from cells by a pH shock: in 10 mL of cell culture, 10 µL of HCl (1 M) was added and distributed over the sample by rapid stirring. Microalgae released flagella upon a change in pH, which was confirmed under an inverted light microscope (Eclipse TE2000-S, Nikon, Tokyo, Japan). Then, 10 µL of NaOH (1 M) was added to the culture to re-establish the initial pH in the culture. SCPs were isolated from a 10 mL sample by differential centrifugation, as described in the next subsection.

### 4.7. Isolation of SCPs from Plant and Microalgae Samples

SCPs were isolated by differential centrifugation, using a protocol widely used for the isolation of small extracellular vesicles [63]. Briefly, the cells were removed by low-speed centrifugation (300× *g*, 10 min, 4 °C, centrifuge Centric 260R with rotor RA 6/50 (Domel, Železniki, Slovenia)), using 50 mL conical centrifuge tubes (ref. S.078.02.008.050, Isolab Laborgeräte GmbH, Eschau, Germany); and 2000× *g*, 10 min, 4 °C (Centric 400R centrifuge with rotor RS4/100 (Domel, Železniki, Slovenia)), using 15 mL conical centrifuge tubes (ref. S.078.02.001.050, Isolab Laborgeräte GmbH, Eschau, Germany). Each step was repeated twice. Then, the cell-depleted medium was centrifuged twice at 10,000× *g* and 4 °C for 30 min (Beckman L8-70M ultracentrifuge, rotor SW55Ti (Beckman Coulter, Brea, CA, USA)), using thin-wall polypropylene centrifuge tubes (ref. 326819, Beckman Coulter, Brea, CA, USA) to remove larger cell debris. Finally, NPs were pelleted by ultracentrifugation at 118,000× *g* and 4 °C for 70 min in the same type of ultracentrifuge and ultracentrifuge tubes. The pellet was resuspended in 50 µL of the initial medium (PBS/ultraclean waste/marine water).

### 4.8. Isolation of SCPs from Washed Erythrocytes

On day 6 after blood collection, the erythrocyte suspension was homogenized by gently inverting the tube 5–10 times. Samples were then subjected to sequential centrifugation of supernatants for 10 min at 500× *g*, 2000× *g*, and 4000× *g*, all at 4 °C, in the centrifuge Centric 400/R (Domel, Železniki, Slovenia). After the last step, the supernatant was subjected to centrifugation at 50,000× *g* and 4 °C for 70 min in an ultracentrifuge Beckman L8–70M with a rotor SW55Ti (Thermo Fisher Scientific, Waltham, MA, USA). The pelleted vesicles were resuspended in 5 mL of PBS-citrate and centrifuged at 50,000× *g* at 4 °C for 70 min. The pellet was resuspended in 1 mL PBS-citrate to obtain the isolate, which was stored at 4 °C until analyzed.

### 4.9. Isolation of SCPs from Plasma

Two hundred and fifty microliter aliquots of plasma were placed in Eppendorf tubes and centrifuged for 10 min at 17,570× *g* and room temperature in the Centric 200R centrifuge with Lilliput rotor (Domel, Železniki, Slovenia). Upper 200 microliters of supernatant were replaced with PBS, resuspended and centrifuged again for 10 min at 17,570× *g* and room temperature in the Centric 200R centrifuge with Lilliput rotor (Domel, Železniki, Slovenia). Two hundred microliters of supernatant were removed, and the pellet was suspended in 80 microliters of PBS.

### 4.10. Preparation of Liposomes

Liposomes were prepared by mixing appropriate proportions of the lyophilized soya lecithin granules with ultraclean water/supernatant of isolation of SCPs from spruce needle homogenate and glycerol at room temperature [64]. Three samples were prepared. Sample A contained 25 weight% of soya granules, 50% of water, and 25% of glycerol; sample B contained 25 weight% of soya granules, 50% of the supernatant of the isolation of SCPs from spruce needle homogenate and 25% of glycerol, and sample C contained equal weight% of soya granules, supernatant of isolation of SCPs from spruce needle homogenate, and glycerol. In cases A and B, soyabean lecithin granules were placed into the falcon tubes. Water/supernatant was added, and the suspension was left at room temperature for 1 h. Glycerol was added, and the samples were mixed by pipetting with a Pasteur pipette. In the case of sample C, soyabean lecithin granules were placed into the Falcon tube, and glycerol was added. The sample was mixed by vortexing, and the supernatant from the isolation of SCPs from spruce needle homogenate was added. The sample was mixed by turning the Falcon tube upside down. The sample was kept at room temperature for 6 months. Before characterization, all three samples were placed on a carousel mixer for 1 day. Two dilutions (100× and 1000×) were prepared, filtered through 0.4 μm filters and divided into aliquots to be measured by different methods.

### 4.11. Cryogenic Electron Microscopy (Cryo-TEM)

C-flat™ 2/2, 200 mesh holey carbon grids (Protochips, Morrisville, NC, USA) were glow discharged at 20 mA for 60 s in a positive polarity air atmosphere (GloQube ^®^ Plus, Quorum, Laughton, UK). Three microliters of sample were applied to the grid, blotted, and vitrified in liquid ethane on the Vitrobot Mark IV (Thermo Fisher Scientific, Waltham, MA, USA). Vitrobot conditions were set to 100% relative humidity at 4 °C, a blot force of 2, and a blot time of 7 s. Samples were visualized under cryogenic conditions using a 200 kV Glacios microscope with a Falcon 3EC detector (Thermo Fisher Scientific, Waltham, MA, USA).

### 4.12. Flow Cytometry (FCM)

The particle numbers in samples from liposomes, blood, spruce homogenate, and microalgae *Phaeodactylum tricornutum* were estimated by flow cytometry [65] using a MACSQuant Analyzer flow cytometer (Miltenyi Biotec, Bergisch Gladbach, Germany) and the related software. The following instrument settings were employed: FSC: 458 V; SSC: 467 V with a trigger set to 1.48; B3: 300 V; and R1: 360 V. Particles were detected from the forward (FSC) and side scatter parameters (SSC). The particle numbers in samples from the microalgae *Tetraselmis chuii* were estimated by an Aurora spectral flow cytometer (Cytek, Biosciences, Fremont, CA, USA) using an excitation laser of 488 nm. Samples were mixed by pipetting before measurement, and 20,000 events per well were acquired. Data were analyzed using Aurora software or FlowJo software (BD Biosciences, Franklin Lakes, NJ, USA).

### 4.13. Ultraviolet Spectroscopy (UV-VIS)

Three microliters droplets of samples were assessed by a nanodrop UV spectrometer (Thermofischer Scientific, Waltham, MA, USA) applying ultraviolet-visible spectrum (UV-Vis) absorbance. The wavelength 280 nm was considered in all samples, while the wavelength 420 nm was considered in blood-derived samples. Samples were vortexed before measurement.

### 4.14. Dynamic Light Scattering (DLS)

The average hydrodynamic radius (R_h_) of NPs and the average intensity of light (I) scattered from NPs in the samples were assessed by Dynamic Light Scattering (DLS). The value of I was interpreted as a measure of NP number density (in the case of preserved particle size distribution) or as a topological change (in the case of altered R_h_ distribution). The correlation functions and integral time-averaged intensities I(θ) ≡ I(q) (where q is the scattering vector, defined as q = (4πn_0_/λ_0_) sin(θ/2), with n_0_ the refractive index of the medium, in our case estimated by the corresponding value for water, i.e., n_0_ = 1.33 at 25 °C), were recorded simultaneously. The R_h_ values of SCPs were obtained from the diffusion coefficients (D) that were assessed from the correlation function of the scattered electric field (g_1_(t)). The g_1_(t) function was calculated from the measured correlation function of the scattered light intensity g_2_(t) applying Siegert’s relation. To convert D to R_h_, the Stokes–Einstein equation was used (R_h_ = kT/6πηD, where k is the Boltzmann constant, T is the absolute temperature, and η is the viscosity of the medium in which the particles diffuse). The Stokes–Einstein equation is strictly valid only for particles of a spherical shape. The viscosity of the medium was approximated to the viscosity value of water at 25 °C. For analysis of the samples from liposomes, blood, spruce homogenate, and microalgae *Tetraselmis chuii* and *Phaeodactylum tricornutum*, we used the Instrument 3D-DLS-SLS cross-correlation spectrometer from LS Instruments GmbH (Fribourg, Switzerland) with a 100 mW DPSS laser (Cobolt Flamenco, Cobolt AB, Solna, Sweden) having a wavelength λ_0_ = 660 nm. Before measurements, samples were equilibrated in a decalin bath at 25 °C for 15 min. The scattered light was measured at an angle of θ = 90° for 120 s. The analysis was carried out using in-house-created software based on the inverse Laplace transform program CONTIN. The in-house software performs the same functions as the program CONTIN. On 25 January 2011, the code for the program CONTIN (which is freely available online) was accessed. We collected several intensity correlation functions for each setting. Curves were analyzed independently and compared with the averaged curve. The correlation curves were fitted with up to 50 exponents. More details on DLS measurements can be found in [66].

### 4.15. Interferometric Light Microscopy (ILM)

**Figure 4:**
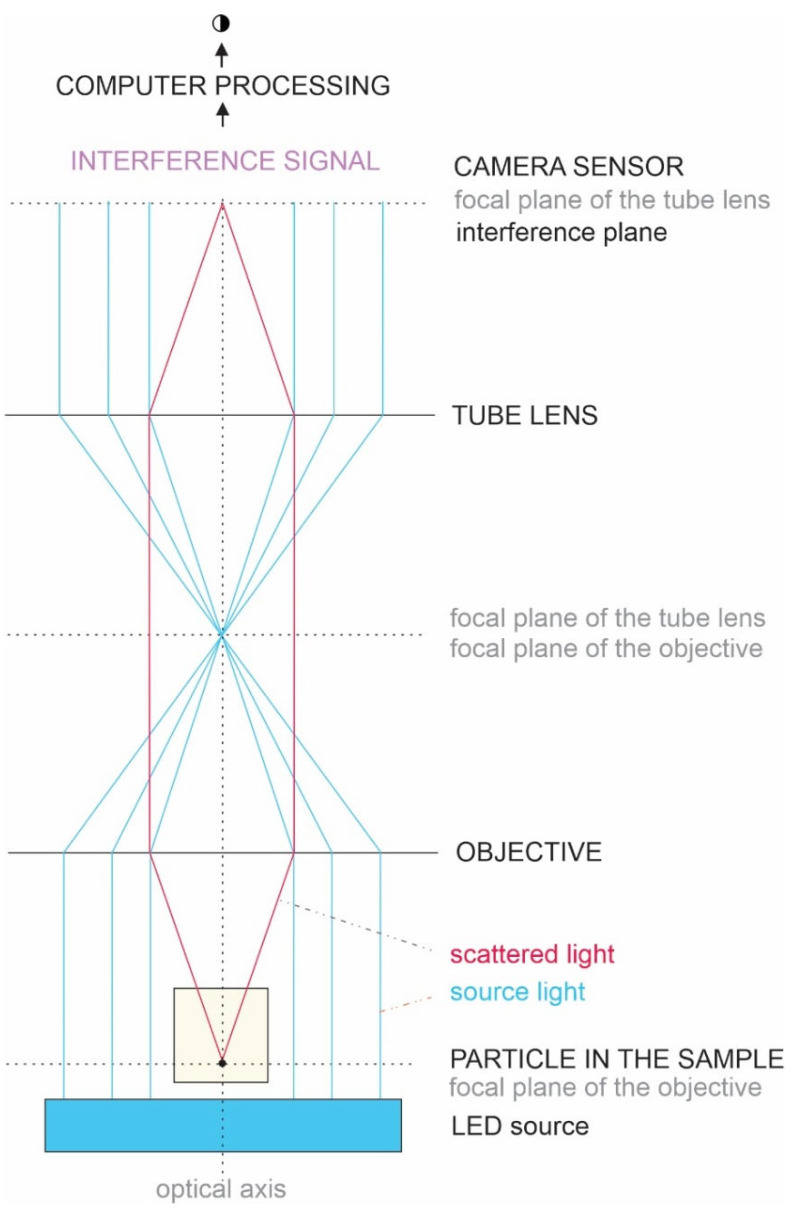
Scheme of ILM.

The average hydrodynamic radius (R_h_) and the number density of SCPs were determined by ILM using Videodrop (Myriade, Paris, France). Signals from the media (ultraclean water, PBS, and marine water) were under the detection limit. The threshold value of 4.2 was used. Seven microliters of sample were placed between cover glasses and illuminated by 2 W of blue LED light. The light scattered on the particle was imaged by a bright-field microscope objective and allowed to interfere with the incoming light (Figure 4). The image was recorded by a complementary metal-oxide-semiconductor high-resolution high-speed camera. Interference enhances the information in the scattered light. However, although the intensity of the interference signal is about 3 orders of magnitude higher than the intensity of the scattered light, the intensity of the incident light is about 3 orders of magnitude higher than the intensity of the interference signal. Therefore, the contribution of the incident light was subtracted from the detected image. The obtained pattern, which includes contrasting black and white spots, was recognized as a particle, and its position in the sample was assessed. The number density of the particles is the number of detected particles within the detected volume, which depends on the microscope characteristics and the particles’ size. The typical detection volume was 15 pL. R_h_ was estimated by tracking the position of the imaged particle within the recorded movie. It was assumed that particles undergo Brownian motion due to collisions with surrounding particles. The motion is random, but the kinetic energies and momenta of the particles reflect the temperature of the sample. Particles with smaller masses move within a larger volume than particles with larger masses. The diffusion coefficient D of the motion of the particle is taken to be proportional to the mean square displacement d of the particle between two consecutive frames taken in the time interval ∆t, <d^2^(∆t)> = <4D ∆t>, while the hydrodynamic radius was estimated by assuming that the particles were spherical and using the Stokes–Einstein relation R_h_ = kT/6πηD. Each particle that was included in the analysis was tracked and processed individually, and the respective incident light signal was subtracted from each image. Processing of the images and the movies was performed by using the associated software, QVIR 2.6.0 (Myriade, Paris, France).

The experimental error in determining number density and R_h_ in selected samples from four natural sources and liposomes was estimated (Table 4). Three measurements were made, and the largest difference from the mean was taken as the error. The spruce homogenate samples showed the largest error with respect to both number density and R_h_.

## 5. Conclusions

Despite many methods for EP characterization, current knowledge on SCPs is rudimentary, and elucidation of their morphology, size, and number density remains a challenge. We have applied four different batch methods and cryo-TEM to five different types of nanoparticles in 30 samples. Decisive information was obtained by all methods for blood-derived samples, spruce needle homogenate, and liposomes. ILM showed excellent agreement with DLS and cryo-TEM in the determination of R_h_ in globular colloidal vesicles (SCPs from blood and liposomes) and was the only method used here that yielded their number density. Cryo-TEM revealed that the shape of flagellosomes was not globular. In both methods, flagellosomes would be better assessed by the diffusion coefficient than by R_h_. Cryo-TEM revealed the presence of thin filaments, which could have resulted in an overestimation of R_h_ by ILM and DLS in *Phaeodactylum tricornutum* isolates. We discovered that ILM is a useful method for estimating the number density and size of SCPs in samples of various origins. The advantages of ILM are its small sample volume requirement, minimal invasiveness, and time effectiveness. While complementary information from batch methods proved useful in the characterization of the samples, visualization of the sample content by cryo-TEM is key to understanding its contents and in interpreting the results of the batch methods.

## Figures and Tables

**Table 1:** Amount of material in samples from blood (B), spruce homogenate (S), flagellae of *Tetraselmis chuii* (T), conditioned culture media of *Phaeodactylum tricornutum* (P), and liposomes (L) as detected by four methods (FCM, ILM, DLS, and UV-vis).

	FCM		ILM		DLS *				UV-vis	
									280 nm	420 nm
Sample	Dilution *	N/mL	Dilution	N/mL	I_tot_ (kHz/mW)	I_1_/I_tot_	I_2_/I_tot_	I_3_/I_tot_	(mg/mL)	(mg/mL)
B1/IS	70×	3.9 × 10^8^	40	4.8 × 10^10^/LP					25.1	13.8
B2/IS			10	1.8 × 10^10^	411 ^#^	0.01	0.63	0.27	4.70	
B3/SN		1.0 × 10^6^	10	1.4 × 10^10^	563	0.17	0.62	0.22	19.6	1.02
B4/SN		3.6 × 10^5^		1.3 × 10^9^	24 ^#^	0.02	0.36	0.53	0.87	0.01
B5/IS		6.6 × 10^6^	50x	1.8 × 10^11^	1681	0.22	0.78		1.54	1.53
B8/IS		3.3 × 10^5^		3.4 × 10^9^					0.9	2.74
S1/IS				UDL					25	
S6/IS		2.1 × 10^5^		UDL					25	
S9/IS				2.8 × 10^8^					24	
S11/IS			3x							
S12/IS				UDL					27	
S13/IS			10x							
T1/IS		4.4 × 10^7^		9.6 × 10^8^	70				0.09	
P1/IS		3.6 × 10^8^							0.13	
P2/IS		6.7 × 10^5^							0.08	
P3/IS		7.1 × 10^5^		6.3 × 10^8^/B	17				1.25	
P4/IS		3.6 × 10^5^		4.0 × 10^8^/LP	5.2				1.19	
P11/IS		3.9 × 10^5^		2.1 × 10^8^/LP					0.16	
P12/IS		3.8 × 10^4^		3.1 × 10^8^/LP					0.19	
P16/IS		7.3 × 10^5^		5.1 × 10^8^/LP/BA					0.37	
P21/SN		2.0 × 10^5^		3.4 × 10^8^/LP					0.19	
P26/SN		7.4 × 10^4^		2.4 × 10^8^/LP/BA					0.88	
LA	1000×	1.3 × 10^9^	5 × 10^4^	9.7 × 10^13^	117	0.05	0.95		370	
LB	1000×	6.3 × 10^8^	2 × 10^4^	9.9 × 10^13^	131	0.27	0.73		340	
LC	1000×	1.7 × 10^9^	2 × 10^5^	1.5 × 10^14^	170	0.01	0.67	0.32	400	
LA	100×		2 × 10^5^	1.1 × 10^14^	961	0.01	0.99		355	
LB	100×		2 × 10^5^	7.8 × 10^13^	973	0.04	0.96		322	
LC	100×		2 × 10^5^	1.1 × 10^14^	1353	0.06	0.67	0.26	378	

* Results for FCM, ILM, and UV-vis are corrected for factors of dilution. N: number of particles; I: intensity of scattered light; UDL: under detection limit; BA: presence of bacteria; LP: presence of particles larger than 1 μm; IS: isolate; SN: supernatant. DLS presents I/I_tot_ (with I_tot_ being the total intensity of scattered light) for three detected peaks (I_1_, I_2_, and I_3_ are the corresponding intensities) in the I(R_h_) distribution function. ^#^ In samples denoted by #, the I(Rh) distribution function revealed four peaks (LP: Rh > 10 μm). The estimated error of ILM measurement is given in the Material and Methods Section, Section 4.15.

**Table 2:** Proportions of estimated particle quantity (diluted 1000×/diluted 100×) in liposome samples. Cor-rections due to dilution were taken into account, so ideally, the value 1 would be expected.

Sample	ILM	DLS	UV-vis 280 nm
LA	0.88	1.17	1.04
LB	1.27	1.03	1.06
LC	1.40	1.25	1.05

**Table 3:** Hydrodynamic radii of particles Rh in aliquots of samples B, S, T, P, and L assessed with two methods: ILM and DLS.

	ILM			DLS			
Sample	Dilution	N Tracked	R_h_ ± SD (nm)	Dilution	R_h1_ (nm) (Small)	R_h2_ (nm) (Main)	R_h3_ (nm) (Large)
B1/IS	40×	322	116 ± 36	70×			
B2/IS	10×	320	115 ± 61		10	185	~1000 ^#^
B3/SN	10×	333	68 ± 41		7.6	39	~5000
B4/SN		305	103 ± 56		2.7; 18	120	^#^
B5/IS	50×	381	103 ± 27		70	153	
B6/IS	20×						
B8/IS		350	119 ± 40				
S1/IS		UDL		1000×			~1600 ^#^
S6/IS		UDL					
S9/IS		61	106 ± 79				
S11/IS	3×	9				148	
S12/IS		UDL				142	
S13/IS	10×	1					
T1/IS		184	118 ± 79		18	70	~1000
P1/IS							
P2/IS							
P3/IS		192	193 ± 100			170	
P4/IS		101	194 ± 126			190	
P11/IS		40	199 ± 126				
P12/IS		51	147 ± 93				
P16/IS		166	193 ± 113				
P21/SN		70	143 ± 98				
P26/SN		51	167 ± 122				
LA	5 × 10^4^×	329	152 ± 59	1000×	20	189	
LB	2 × 10^4^×	96	147 ± 59	1000×	71	235	
LC	2 × 10^5^×	138	136 ± 55	1000×	11	122	622
LA	2 × 10^5^×	403	148 ± 54	100×	12	150	
LB	2 × 10^5^×	342	143 ± 61	100×	28	187	
LC	2 × 10^5^×	371	152 ± 64	100×	30	133	413

UDL: under detection limit; BA: presence of bacteria; IS: isolate; SN: supernatant. DLS presents hydrodynamic radii corresponding to 2–4 detected peaks (the column designated by R_h2_ corresponds to the main peak in the size distribution). ^#^ the presence of very large particles (R_h_ > 10 μm). SD: standard deviation. SD is given for samples with at least 40 particles tracked (raw data, DOI: 10.5281/zenodo.7178202).

**Table 4:** Experimental error in selected samples from B, S, P, and L sources.

Sample	Dilution	Mean N/mL^−1^	∆N/Mean N (%)	Mean R_h_ (nm)	∆R_h_/Mean R_h_ (%)
Plasma	50×	2.78 × 10^10^	2	75	1
Erythrocytes	50×	1.33 × 10^11^	6	94	4
Spruce homogenate	5×	5.67 × 10^9^	38	71	12
*P. tricornutum* isolate		1.10 × 10^9^	9	125	11
Liposomes	5 × 10^4^×	1.55 × 10^14^	4	131	4

## Data Availability

The raw data of the ILM analysis are available at DOI: 10.5281/zenodo.7178202.
